# Assessing extraction-analysis methodology to detect fluorotelomer alcohols (FTOH), a class of perfluoroalkyl and polyfluoroalkyl substances (PFAS), in artificial turf fibers and crumb rubber infill

**DOI:** 10.1016/j.cscee.2022.100280

**Published:** 2022-11-30

**Authors:** Philip Zuccaro, James Licato, Emily A. Davidson, David C. Thompson, Vasilis Vasiliou

**Affiliations:** a Yale University, New Haven, CT, USA; b Department of Environmental Health Sciences, Yale School of Public Health, New Haven, CT, USA; c Department of Cellular & Molecular Physiology, Yale School of Medicine, New Haven, CT, USA

**Keywords:** Artificial turf, Crumb rubber, Fluorotelomer alcohol (FTOH), Per-/polyfluoroalkyl substances (PFAS), Gas chromatography-mass spectroscopy (GC-MS)

## Abstract

**Background:**

Despite widespread global use of artificial turf fields, there is a paucity of research assessing the presence of potentially harmful chemicals within the field components.

**Objective:**

This pilot study aimed to assess the capacity of an adapted extraction-analysis method to identify and quantitate FTOHs, a class of perfluoroalkyl and polyfluoroalkyl substances (PFAS), in artificial turf fiber and crumb rubber infill samples.

**Methods:**

FTOHs in artificial turf fibers and crumb rubber infill were extracted using 80:20 methanol:methyl *tert-*butyl ether, reconstituted in methanol, and analyzed by gas chromatography-mass spectroscopy (GC-MS) operated in scanning ion mode (SIM).

**Results:**

8:2 FTOH was detected in artificial turf fiber and crumb rubber infill samples at concentrations of 1.0 and 0.71 ng/μL, respectively. This translates to 300ng 8:2 FTOH/g artificial turf fiber and 110ng 8:2 FTOH/g crumb rubber. By contrast, 4:2 FTOH and 6:2 FTOH were not found to be present in detectable levels.

**Conclusion:**

Our extraction method with subsequent GC-MS analysis proved useful in detecting FTOHs in artificial turf field samples. 8:2 FTOH may be present in artificial turf fibers and crumb rubber infill. This pilot investigation supports the need for further research into the presence of this class of PFAS in artificial turf field components.

## 1. Introduction

As many as 13,000 artificial turf fields are utilized across the United States [1]. The European Chemicals Agency (ECHA) estimates that there are at least 13,000 full-length artificial turf fields and 45,000 smaller-sized fields in use in the European Union (EU) [2]. Many of the regular users of these fields are children, adolescents, or young adults.

**Fig. 1.**
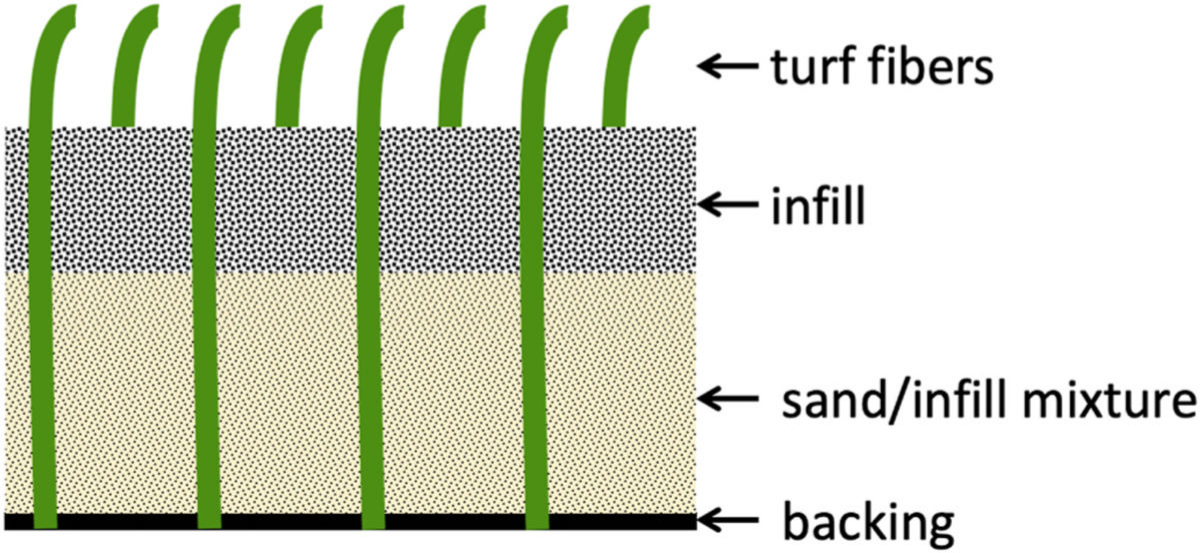
[4] Artificial turf field components. The turf backing acts as a base connection point for the turf fibers, while the infill and sand/infill mixture layers are utilized to provide structural support.

The primary components of artificial turf fields include the turf fibers, infill, and backing. The turf fibers are the upper layer of individual “grass” blades, the infill is the layer of granulated substance that holds the fibers upright and provides cushioning, and the backing is the bottom layer to which the fibers are connected and on which the infill rests (Fig. 1) [3,4]. Crumb rubber granules are by far the most widely used infill substance [2]. They are created by shredding recycled end-of-life tires, and thus contain chemicals from which the original tires were made.

Despite widespread use of artificial turf fields, limited research has been conducted to investigate the presence of potentially harmful chemicals in and health risks of their constituent components. Crumb rubber alone contains hundreds of chemical agents, including polycyclic aromatic hydrocarbons (PAHs), volatile organic compounds (VOCs), polybrominated substances, and heavy metals such as lead and zinc [5]. Most constituent chemicals, however, have not been catalogued by the United States Environmental Protection Agency (US EPA) [5]. Thus, reliable information about the potential health effects of the majority of these components is not available.

A chemical group of potential concern in artificial turf fields is the perfluoroalkyl and polyfluoroalkyl substances (PFAS). Many of these chemicals are carcinogenic, elicit immunotoxic effects, harm the endocrine system, or compromise reproductive health [6–8]. In addition, PFAS are known as “forever chemicals” due to their permanence and long-term accumulation in the environment and in biological systems (such as the human body) [4,6,7]. Due to their adverse health effects, the production of several PFAS chemicals, such as perfluorooctanoic acid (PFOA), has been restricted or banned outright in many countries [4]. However, many PFAS remain in use worldwide. These include the fluorotelomer alcohols (FTOHs), a class of PFAS known to be volatile precursors of other, more harmful PFAS such as PFOA [9]. In animals, the metabolic conversion to PFOA has been shown to occur rapidly, while similar conversion may also occur in humans [10–12]. It has been demonstrated, for example, that levels of FTOH in building air predict serum levels of PFOA in office workers [13]. It is therefore possible that exposure to FTOHs could lead to adverse health effects through the endogenous generation of PFOA.

Based on the current paucity of research concerning FTOHs in artificial turf fields and the potential health risks posed by exposure to these chemicals, our study aimed to assess the utility of an FTOH extraction-analysis method adapted for artificial turf fibers and crumb rubber infill. In doing so, our pilot investigation also provides an initial assessment of the presence of FTOHs (specifically 4:2, 6:2, and 8:2 FTOH) in a single sample of artificial turf field components.

## 2. Methods

A single sample of new artificial turf fibers and crumb rubber pellets was obtained from an artificial turf field product distributor in fully sealed packaging. The choice of sample was based on the availability of products representing an average artificial turf field with corresponding crumb rubber infill. The sample remained sealed in the original packaging, shielded from sunlight and extreme temperatures until the time of data collection. All reagents were analytical grade and obtained from Sigma-Aldrich (USA). The 4:2, 6:2, and 8:2 FTOH utilized for gas chromatography-mass spectroscopy (GC-MS) standards were purchased from Thomas Scientific (New Jersey, USA).

An FTOH extraction method for artificial turf field components was devised by utilizing aspects of several existing methods used for biological specimens and textile products [14–16]. Individual artificial turf fibers were cut from the turf backing using scissors (≈2.5 cm original length). Turf fibers (5 g) were placed into plastic conical tubes with 30 mL methanol:methyl *tert*-butyl ether (80:20) and subjected to sonication for 30 minutes. The sample then underwent centrifugation (Savant SPD111V) at ambient temperature for 148 minutes. The resulting supernatant (0.25 mL) was transferred to a plastic conical tube and all samples were stored at 4°C. The same procedures were used for extraction from crumb rubber infill pellets, except that 10 g of crumb rubber pellets were placed in the plastic conical test tubes with 30 mL methanol:methyl *tert*-butyl ether (80:20).

For GC-MS analysis of FTOHs, each sample was reconstituted in 1.5 mL methanol in an autosampler vial. The samples (10 μl injection volume) were applied to the GC-MS system (Agilent Technologies 7890B GC with MassSpec 5977 and 7693A Automatic Liquid Sampler) using a 30 m DB-Wax column (0.25 μm film thickness x 0.25 mm inner diameter, Agilent). Analyses were conducted using positive chemical ionization (PCI). The GC oven schedule adhered to that used previously by Butt et al. [15] A starting temperature of 60°C was maintained for 1 min, then increased 5°C/minute until the system reached 75°C, followed by a 10°C/min increase until reaching 130°C, and finally a 50°C/min increase to 240°C, which was maintained for 1 min. Two replicates of identical volume and content were run for each sample, and each replicate was run three separate times to account for variability and insufficient column optimization.

A working mix containing 4:2, 6:2, and 8:2 FTOH analytes was generated and utilized to create five-point calibration standards (1, 5, 10, 25, and 50 ng/μL) for GC-MS analysis. One replicate of the 10 g crumb rubber sample was combined with the working mix to serve as a matrix spiked sample. The identification of 4:2, 6:2, and 8:2 FTOH presence in the samples was based on the peak comparisons from the resulting GC-MS selected ion monitoring (SIM) spectra for each respective chemical, compared to that of the calibration standards and matrix-spiked sample. The mass-to-charge (*m/z*) ratios selected for analysis were 264.02, 364.01, and 464.01 for 4:2, 6:2, and 8:2 FTOH, respectively.

## 3. Results

**Fig. 2.**
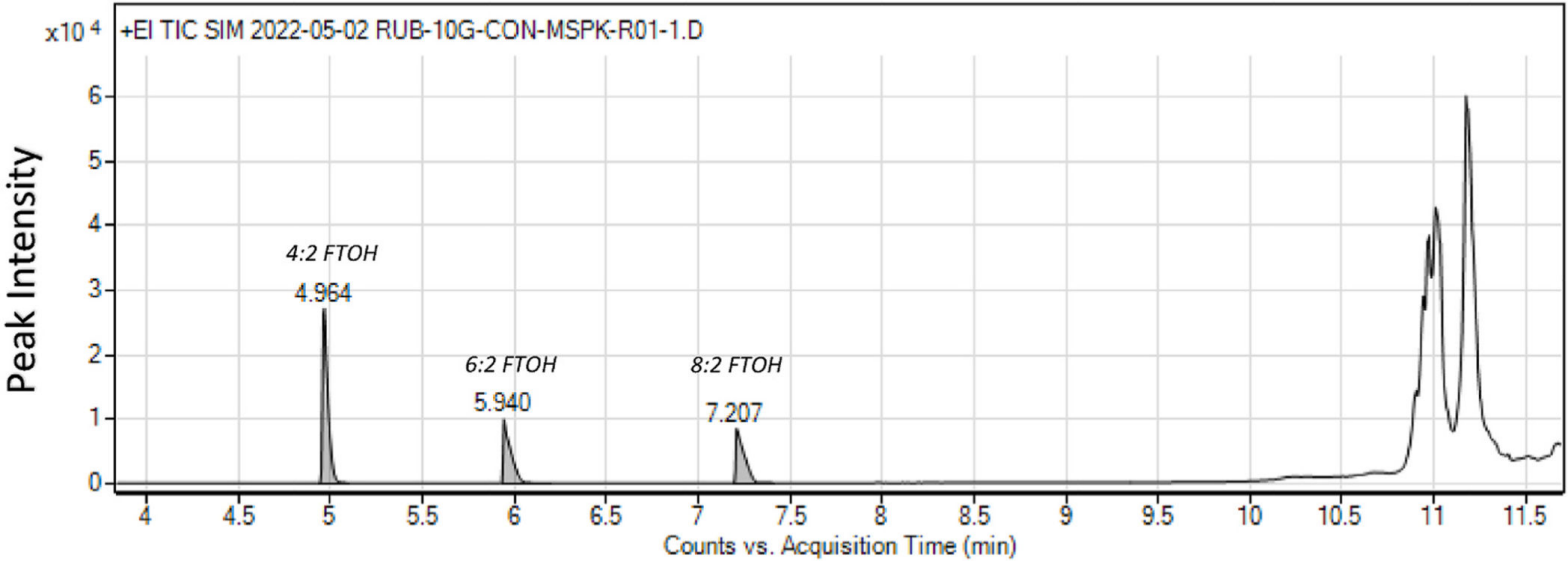
Representative integrated GC-MS spectrum for a crumb rubber matrix spike sample.

The resulting spectrum from the matrix spiked sample aligned with that of the calibration standards. For the crumb rubber matrix spike samples, the average acquisition times for peaks corresponding to 4:2, 6:2, and 8:2 FTOH were 5.0, 5.9, and 7.2 minutes, respectively. This matches that of the expected peaks in the calibration standards, which showed average acquisition times of 5.0, 6.0, and 7.3 minutes. A representative first run recorded spectrum for the crumb rubber matrix spike samples is shown in Fig. 2. These results provide validation of our extraction and GC-MS methods.

The area under the curve for detected peaks (i.e., integration value) in the GC-MS spectra was used to estimate FTOH levels. Five known concentrations of the FTOHs were subjected to linear regression with forced-through-zero function [17] (GraphPad Prism) to generate standard calibration curves for each of the FTOHs. The high correlation coefficients (0.99) for each curve suggest that our GC-MS method was functional and able to detect the three FTOHs in samples (Table 1). The 10 g crumb rubber matrix spike recorded average percent yields of 642.3%, 94.5%, and 81.9% for 4:2, 6:2, and 8:2 FTOH, respectively. Levels of FTOH in turf fiber or crumb rubber samples were determined by interpolation of peak areas on the standard calibration curves and were corrected based on these percent yield values. Lower limits of detection (LLOD) and lower limits of quantification (LLOQ) were calculated for each FTOH as a measure of the GC-MS method sensitivity.

Concentrations of the FTOHs in samples derived from crumb rubber and artificial turf fibers were found to be consistent between runs and replicate samples (Table 2). The only exception related to 6:2 FTOH’s being detected (0.49 ng/μL) in the first run of the first crumb rubber sample replicate. Due to its absence from all other runs and replicates (i. e., <LLOD), we considered 6:2 FTOH not to be present in the samples. Only 8:2 FTOH concentrations exceeded the lower limits of detection and quantification, with the average levels in crumb rubber and artificial turf being 0.71 and 1.0 ng/μL, respectively. Taking into account the amount of source sample (i.e., turf fiber or crumb rubber), this translates to 300 ng 8:2 FTOH/g artificial turf fiber and 110 ng 8:2 FTOH/g crumb rubber.

## 4. Discussion

This is the first study to establish an extraction-analysis method to detect FTOHs, a class of PFAS, in artificial turf fibers and crumb rubber infill. Our GC-MS analyses detected 8:2 FTOH to be present in both artificial turf fiber and crumb rubber samples. Concentrations of 4:2 and 6:2 FTOH were found to be below the lower limits of detection. Our extraction-analysis method proved to be functional for detecting FTOHs in samples, and demonstrated the capacity to distinguish the presence of different FTOHs.

Notably, the concentration of 8:2 FTOH in the artificial turf fibers exceeded that of the crumb rubber infill. When taking into account the relative amount of turf fiber or crumb rubber from which samples were obtained, there was almost three times as much 8:2 FTOH associated with turf fibers (300 ng/g fiber) than crumb rubber (110 ng/g rubber). FTOHs in crumb rubber was less surprising given their source (end-of-life tires) and the many chemicals contained therein. The source of 8:2 FTOH in turf fibers is more difficult to predict. Most, if not all, regulatory attention and health concerns germane to artificial turf fields have been directed at the widely used crumb rubber infill [4]. In response to these concerns, many artificial turf manufacturers have initiated production of alternative infill types such as cork or coated sand. Our results, which need to be validated in subsequent studies using a greater number of original samples sources, suggest that focus should also be placed on the turf fibers.

The potential presence of 8:2 FTOH in components of artificial turf fields is significant, both for the environment and for individual users, as many patrons use the fields on a recurring basis for extended periods of time. Key exposure routes include inadvertent oral ingestion of crumb rubber granules or turf fibers, direct absorption through contact with open wounds, and inhalation of volatilized FTOH, all of which have the potential to lead to endogenous metabolic generation of PFOA [10–13]. As noted, PFOA is toxic to the human body and has consequently been banned in many jurisdictions [4,9–12].

There are multiple limitations to this pilot investigation. First, it is important to appreciate that in the present study, FTOH measurements were made only in a single sample of artificial turf fibers and crumb rubber infill. As such, definitive conclusions regarding the extent of FTOH presence in artificial turf fibers and crumb rubber awaits further studies with a larger number of original samples from different manufacturers. Nevertheless, the most valuable outcome of the present study is the development and validation of analytical procedures that allow for the measurement of FTOHs in such samples. Another limitation is the lack of internal standards utilized in the GC-MS method. Going forward, extraction efficiency should be evaluated by introducing a tagged surrogate of each FTOH standard during the first extraction step. More robust quantification could also have been accomplished by inclusion of a larger number of calibration standards below 50 ng/μL. It should also be noted that the samples used in this study were brand new. Future studies should aim to investigate the extent to which older, used samples contain FTOHs. The lack of inclusion of used crumb rubber samples would be expected to have less impact on study conclusions because crumb rubber is routinely replaced on these fields to maintain infill levels after migration of the pellets into the clothing of field users or to the outside environment. Lastly, establishing the presence of 8:2 FTOH in artificial turf field components does not prove that this chemical enters or accumulates in the bodies of field users, nor has the concentration of 8:2 FTOH detrimental to human health yet been established. Further investigation into the extent of exposure would be critical for full comprehension of the potential risks, if any, to human health.

In conclusion, the present pilot study establishes an extraction-analysis method for detection of FTOHs in artificial turf field components, and provides preliminary evidence that 8:2 FTOH may be present in both artificial turf fibers and crumb rubber infill. These findings support the need for further research into the presence and potential impact of PFAS in artificial turf field constituents.

## Figures and Tables

**Table 1:** Calibration standard curve metrics for 4:2, 6:2, and 8:2 FTOH. Method sensitivity for GC-MS FTOH detection is reported as lower limits of detection (LLOD) and lower limits of quantification (LLOQ), the lowest concentrations at which the chemicals may be considered present in the samples and reliably quantified, respectively.

	Curve Equation^a^	R^b^	LLOD (ng/μL)	LLOQ (ng/μL)
4:2 FTOH	y = 53.1x	0.99	0.36	1.1
6:2 FTOH	y = 25.8x	0.99	0.083	0.25
8:2 FTOH	y = 21.0x	0.99	0.089	0.27

a The equation for the calibration curve reflects the linear regression analysis with forced-through-zero function.

b Correlation coefficient associated with linear regression analysis of standard curve.

**Table 2:** Corrected concentrations for all runs and both replicates of 10 g crumb rubber and 5 g turf fiber samples. Numerical values reported for sample concentrations detected above the lower limits of quantification (>0.25 and > 0.27 ng/μL for 6:2 and 8:2 FTOH, respectively). <LLOD: sample concentrations were below the lower limits of detection (<0.36 and < 0.083 ng/μL for 4:2 and 6:2 FTOH, respectively).

	Run 1	Run 2	Run 3
	
	Corrected Concentration (ng/μL)		
Crumb Rubber (Replicate 1)			
4:2 FTOH	< LLOD	< LLOD	< LLOD
6:2 FTOH	0.49	< LLOD	< LLOD
8:2 FTOH	0.87	0.77	0.77
Crumb Rubber (Replicate 2)			
4:2 FTOH	< LLOD	< LLOD	< LLOD
6:2 FTOH	< LLOD	< LLOD	< LLOD
8:2 FTOH	0.70	0.57	0.57
Turf Fibers (Replicate 1)			
4:2 FTOH	< LLOD	< LLOD	< LLOD
6:2 FTOH	< LLOD	< LLOD	< LLOD
8:2 FTOH	1.10	1.10	0.93
Turf Fibers (Replicate 2)			
4:2 FTOH	< LLOD	< LLOD	< LLOD
6:2 FTOH	< LLOD	< LLOD	< LLOD
8:2 FTOH	0.85	0.90	1.10

## Data Availability

Data will be made available on request.
